# Integrated Fruit Phenotyping and Electronic-Nose Profiling of Five *Ilex* Taxa from Eastern China for Germplasm Characterization and Utilization

**DOI:** 10.3390/plants15101563

**Published:** 2026-05-20

**Authors:** Xiangxian Fan, Qi Tang, Meng Sun, Ye Peng

**Affiliations:** 1 The Co-Innovation Center for Sustainable Forestry in Southern China, College of Life Sciences, Nanjing Forestry University, No. 159 Longpan Road, Nanjing 210037, China; 2 College of Horticulture, Jinling Institute of Technology, Nanjing 210038, China

**Keywords:** *Ilex*, plant genetic resources, germplasm characterization, fruit traits, electronic nose, phenotypic variation

## Abstract

Accurate characterization of closely related *Ilex* taxa is essential for the conservation, documentation, and utilization of plant genetic resources. In this study, five *Ilex* taxa from eastern China (*Ilex rotunda* Thunb., *Ilex chinensis*, *Ilex cornuta* Lindl. & Paxt., *Ilex cornuta* ‘Fortunei’, and *Ilex latifolia* Thunb.) were evaluated using an integrated framework combining fruit morphometric traits, CIELAB color parameters, and electronic-nose (E-nose) volatile fingerprints. Fruit transverse diameter, longitudinal diameter, single-fruit weight, fruit shape index, and peel color traits (L*, a*, b*, and chroma, C*) differed significantly among taxa (one-way ANOVA, all *p* < 0.001). *I*. *cornuta* produced the largest and heaviest fruits, *I*. *chinensis* showed the most elongated fruit shape, and *I*. *rotunda* exhibited the highest redness and chroma values. Chemometric analyses of E-nose responses further improved taxon discrimination and revealed clear divergence in volatile-response patterns. Trait-space relationships were broadly consistent with the preset phylogenetic framework, with *I*. *rotunda* showing the greatest divergence and *I*. *cornuta* and *I*. *cornuta* ‘Fortunei’ showing the closest similarity. These findings indicate that integrated fruit phenotyping and rapid volatile profiling provide a practical approach for *Ilex* germplasm identification, comparative evaluation, and resource documentation, with potential value for conservation planning and horticultural utilization.

## 1. Introduction

The effective conservation and utilization of plant genetic resources depend on accurate characterization and reliable discrimination of taxa maintained in natural populations, living collections, and cultivated landscapes. This is particularly important in species-rich woody genera, in which closely related taxa may show overlapping vegetative features and cultivars, local forms, and allied species are easily confused during collection, documentation, and utilization. The genus *Ilex* L. (Aquifoliaceae) is one of the most species-rich genera of angiosperms and represents the largest assemblage of dioecious woody plants worldwide. Approximately 600 wild *Ilex* taxa are distributed across tropical, subtropical, and temperate regions of both hemispheres, with major centers of diversity in Central and South America and tropical Asia [1]. China is a major center of *Ilex* diversity, harboring 204 taxa mainly distributed on the southern slopes of the Qinling Mountains, throughout the Yangtze River basin, and further south [2]. This diversity provides valuable genetic resources for ornamental use, nectar production, traditional uses, and potential breeding applications.

Many *Ilex* taxa have substantial horticultural and ecological value, yet their identification and comparative evaluation remain challenging. In practice, germplasm documentation often relies heavily on vegetative morphology, which may be insufficient for distinguishing closely related taxa or cultivated forms. This limitation is well illustrated in *Ilex*. A recent genus-wide study integrating nuclear phylogeny, morphology, and distribution showed that several traditional infrageneric groups were not monophyletic, indicating that classifications based mainly on morphology do not always reflect evolutionary relationships in this genus [3]. Likewise, comparative chloroplast genome analysis of 10 widely used *Ilex* species in mid-southern China showed that some taxa are difficult to distinguish using leaf morphology alone, and species-specific chloroplast markers were therefore developed for reliable discrimination [4]. At the population level, a recent microsatellite-based study of *I. chinensis* further demonstrated that clear delimitation is necessary for assessing genetic diversity and for the protection and utilization of *Ilex* germplasm resources [5]. For germplasm management, accurate delimitation is essential because misidentification can compromise accession records, conservation planning, utilization decisions, and taxon-targeted breeding efforts.

In *Ilex*, leaf characters are highly diverse, whereas flowers and fruits are relatively conservative. As a result, traditional classification and practical identification often rely strongly on leaf morphology, while reproductive traits usually provide limited resolution. Fruit traits, however, remain potentially useful because fruit size, shape, weight, and color are visually conspicuous, biologically meaningful, and comparatively easy to quantify. Standardized color systems such as CIELAB coordinates (L*, a*, and b*) and derived chroma values (C*) further enable objective description of fruit appearance and improve comparability among taxa, collections, and studies. Nevertheless, when related taxa overlap in external fruit size and color, visible fruit traits alone may still be insufficient for reliable discrimination.

In addition to visible traits, volatile-associated characteristics may provide complementary information for germplasm characterization [6]. Electronic-nose (E-nose) technology offers a rapid, non-destructive, and relatively low-cost approach for capturing integrated odor-response fingerprints, and it has been increasingly applied in plant material identification, quality evaluation, and chemometric discrimination [7]. Compared with more labor-intensive analytical methods, E-nose profiling may be especially useful as an operationally simple tool for preliminary screening or high-throughput discrimination of related germplasm materials. In *Ilex*, although fruit size and color may be relatively conservative in some related taxa, volatile characteristics may still differ among taxa. Therefore, integrating fruit morphology, colorimetric traits, and volatile-response profiles may help compensate for the limitations of traditional classification based primarily on external morphology.

Despite the rich diversity and utilization potential of *Ilex* in China, comparative studies integrating fruit phenotyping with volatile-response profiling for germplasm characterization remain limited. In particular, closely related taxa and cultivar-like materials require efficient supplementary descriptors to support accession verification, improve documentation in living collections, and facilitate the evaluation of materials for horticultural use. Developing a practical and non-destructive framework is therefore important not only for taxonomic discrimination but also for applied germplasm management and utilization.

**Figure 1:**
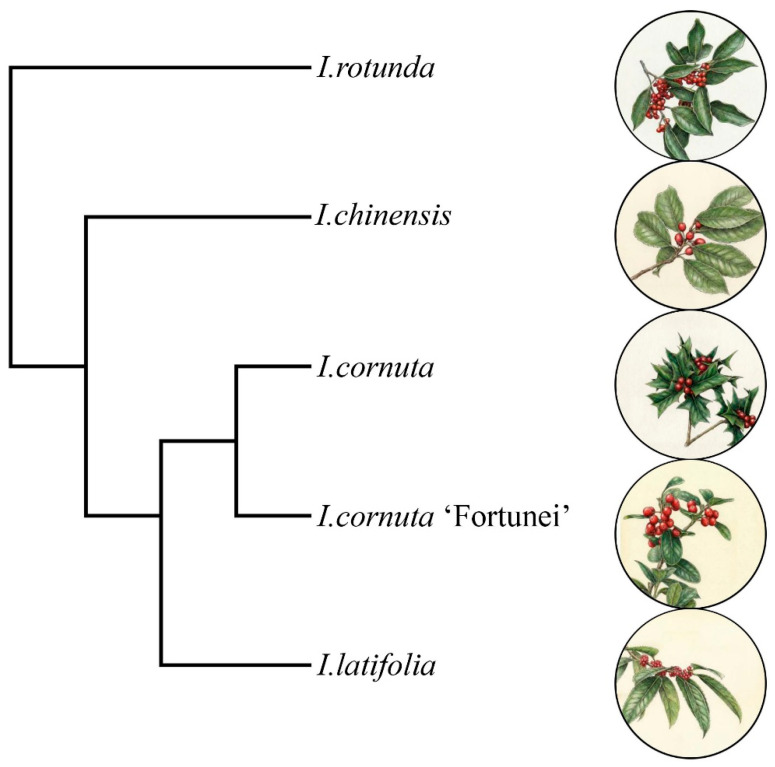
Simplified phylogenetic relationships of the five *Ilex* taxa used for comparative germplasm evaluation, compiled from published phylogenies.

In this study, we used an integrated and non-destructive framework combining fruit morphometric measurements, CIELAB color analysis, and PEN3 electronic-nose profiling to characterize five representative *Ilex* taxa from eastern China, namely *I. rotunda*, *I. chinensis*, *I. cornuta*, *I. cornuta* ‘Fortunei’, and *I. latifolia*. We also compared the observed patterns of trait differentiation with a simplified phylogenetic framework (Figure 1), which was compiled and independently illustrated by the authors based on previously published phylogenies of the genus *Ilex* [8,9]. In *Ilex*, leaf morphological traits are highly diverse, while flower and fruit traits are relatively conservative with low phenotypic variation. Fruit size and color traits are rather uniform and have low resolution for species discrimination in traditional taxonomy. It has been well documented that leaf volatile components differ distinctly among *Ilex* species. However, whether fruit volatiles exhibit similar interspecific differentiation remains unclear. This study hypothesized that electronic nose detection, as a convenient and efficient method, can capture fruit odor-related volatile profiles and identify characteristic volatile markers to effectively distinguish closely related *Ilex* taxa.

## 2. Results

### 2.1. Variation in Fruit Morphometric and Colorimetric Traits Among Ilex Taxa

All measured fruit traits differed significantly among the five *Ilex* taxa, indicating marked intertaxon variation in fruit size, shape, weight, and peel coloration. Table 1 summarizes eight quantitative traits, including transverse diameter (TD), longitudinal diameter (LD), single-fruit weight (FW), fruit shape index (FSI = LD/TD), CIELAB coordinates (L*, a*, b*), and chroma (C*). Different lowercase letters following the mean values denote significant pairwise differences among taxa (LSD post hoc test, *p* < 0.05).

**Figure 2:**
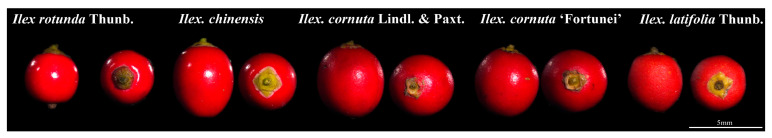
Representative mature fruits of five *Ilex* taxa. Lateral and apical views are shown for each taxon to illustrate differences in fruit size, shape, and peel coloration. Core color characteristics (mean CIELAB values) for each taxon are listed below: *I. rotunda* (L* = 41.82, a* = 52.90, C* = 62.70); *I. chinensis* (L* = 45.26, a* = 46.89, C* = 55.83); *I. cornuta* (L* = 42.58, a* = 48.23, C* = 56.28); *I. cornuta* ‘Fortunei’ (L* = 43.26, a* = 45.32, C* = 52.75); *I. latifolia* (L* = 40.55, a* = 35.92, C* = 42.21).

Representative photographs of mature fruits are shown in Figure 2, providing visual documentation of intertaxon differences in fruit size, shape, and peel coloration.

#### 2.1.1. Fruit Size and Weight

Fruit dimensions differed significantly among taxa (Table 1). *I. cornuta* produced the largest fruits (TD, 7.62 ± 0.51 mm; LD, 8.83 ± 0.62 mm), significantly exceeding those of the other taxa. *I. chinensis* had the second-largest fruits (TD, 7.09 ± 0.48 mm; LD, 8.50 ± 0.57 mm). In contrast, *I. rotunda* had the smallest fruits (TD, 5.64 ± 0.39 mm; LD, 5.86 ± 0.41 mm). The remaining taxa showed intermediate values: *I. cornuta* ‘Fortunei’ (TD, 6.79 ± 0.43 mm; LD, 7.91 ± 0.53 mm) and *I. latifolia* (TD, 6.84 ± 0.45 mm; LD, 7.08 ± 0.49 mm), with significant differences among taxa indicated by the post hoc lettering in Table 1. Single-fruit weight differed significantly among taxa (*p* < 0.001), ranging from 0.183 ± 0.019 g in *I. latifolia* to 0.313 ± 0.035 g in *I. cornuta* (Table 1). *I. cornuta* had the highest FW (0.313 ± 0.035 g), followed by *I. chinensis* (0.231 ± 0.028 g). *I. rotunda* (0.203 ± 0.022 g) and *I. cornuta* ‘Fortunei’ (0.196 ± 0.021 g) formed an intermediate-low group, whereas *I. latifolia* had the lowest FW. Notably, FW did not strictly mirror the ranking of fruit dimensions across taxa: although *I. latifolia* showed intermediate TD and LD, it had the lowest mass, indicating that fruit size and mass were not perfectly coupled among the five taxa.

**Figure 3:**
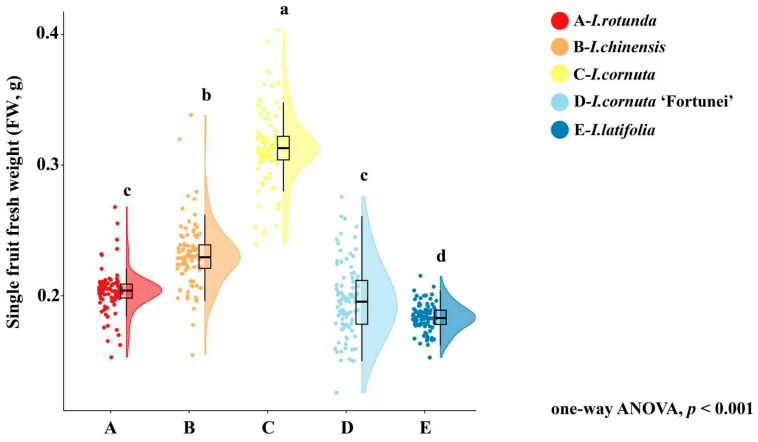
Variation in single-fruit fresh weight among five *Ilex* taxa. A, *I. rotunda*; B, *I. chinensis*; C, *I. cornuta*; D, *I. cornuta* ‘Fortunei’; E, *I. latifolia*. FW indicates single-fruit fresh weight. Each point represents one individual fruit measurement. The half-violin plots show the distribution density of the data, and the box plots indicate the median and interquartile range. Whiskers represent the data range excluding extreme outliers. Different lowercase letters above the plots indicate significant differences among taxa according to one-way ANOVA followed by LSD post hoc multiple comparison test at *p* < 0.05. The overall ANOVA result was significant at *p* < 0.001. n = 90 fruits per taxon.

The raincloud plot of FW (Figure 3) showed that *I. cornuta* shows the highest central tendency (median ≈ 0.31 g), with most observations concentrated at higher FW values, whereas *I. latifolia* exhibits a lower and relatively narrow distribution centered at approximately 0.18–0.19 g. *I. cornuta* ‘Fortunei’ showed a broader violin and longer tails, suggesting greater within-taxon variability in fruit mass than the other taxa. Occasional high-FW observations are also evident in *I. cornuta*, consistent with its higher mean FW reported in Table 1.

#### 2.1.2. Fruit Shape Index (FSI)

FSI (LD/TD) differed significantly among taxa (*p* < 0.001), indicating variation in fruit elongation (Table 1). *I. chinensis* exhibited the highest FSI (1.200 ± 0.062), indicating the most elongated fruits. *I. cornuta* (1.161 ± 0.055) and *I. cornuta* ‘Fortunei’ (1.169 ± 0.058) formed an intermediate group. By contrast, *I. rotunda* (1.038 ± 0.049) and *I. latifolia* (1.036 ± 0.047) had the lowest FSI, consistent with a more nearly spheroidal fruit shape.

**Figure 4:**
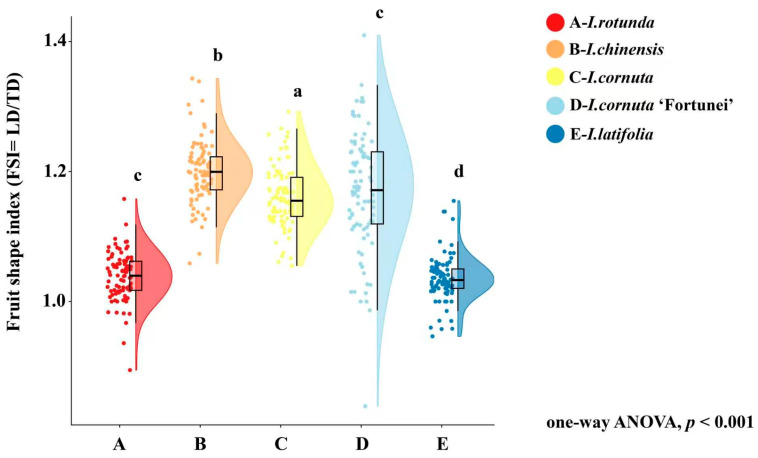
Variation in fruit shape index among five *Ilex* taxa. A, *I. rotunda*; B, *I. chinensis*; C, *I. cornuta*; D, *I. cornuta* ‘Fortunei’; E, *I. latifolia*. Fruit shape index, FSI, was calculated as the ratio of longitudinal diameter to transverse diameter, FSI = LD/TD. LD indicates fruit longitudinal diameter, and TD indicates fruit transverse diameter. Each point represents one individual fruit measurement. The half-violin plots show the distribution density of FSI values, and the box plots indicate the median and interquartile range. Whiskers represent the data range excluding extreme outliers. Different lowercase letters above the plots indicate significant differences among taxa according to one-way ANOVA followed by LSD post hoc multiple comparison test at *p* < 0.05. The overall ANOVA result was significant at *p* < 0.001. n = 90 fruits per taxon.

The FSI raincloud plot (Figure 4) corroborates these group-level differences while highlighting within-taxon dispersion. *I. chinensis* shows an upward-shifted distribution centered at approximately 1.20 with relatively concentrated density, indicating consistently elongated fruits. *I. rotunda* and *I. latifolia* cluster tightly around 1.03–1.05, suggesting relatively uniform fruit shape. By contrast, *I. cornuta* ‘Fortunei’ exhibits a broader violin and longer tails, indicating greater within-taxon heterogeneity in FSI and occasional extreme values, although its mean FSI remains within the intermediate group (Table 1).

#### 2.1.3. Color Parameters

Colorimetric traits differed markedly among taxa (all *p* < 0.001; Table 2). Lightness (L*) was highest in *I. chinensis* (45.26 ± 2.31) and lowest in *I. latifolia* (40.55 ± 2.18). Redness (a*) showed the strongest differentiation, with *I. rotunda* had the highest a* (52.90 ± 3.25), whereas *I. latifolia* had the lowest (35.92 ± 2.87). Yellowness (b*) followed a similar trend, with *I. rotunda* highest (33.24 ± 2.98) and *I. latifolia* lowest (22.16 ± 2.53). Consequently, chroma (C*), which reflects overall color saturation, was highest in *I. rotunda* (62.70 ± 3.52) and lowest in *I. latifolia* (42.21 ± 3.15). *I. chinensis* (55.83 ± 3.09) and *I. cornuta* (56.28 ± 3.12) formed an intermediate group, whereas *I. cornuta* ‘Fortunei’ (52.75 ± 3.15) showed lower chroma.

**Figure 5:**
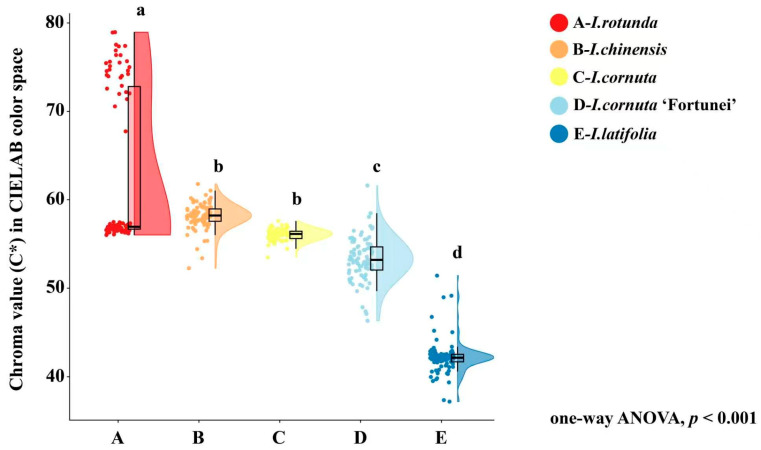
Variation in fruit peel chroma among five *Ilex* taxa based on the CIELAB color system. A, *I. rotunda*; B, *I. chinensis*; C, *I. cornuta*; D, *I. cornuta* ‘Fortunei’; E, *I. latifolia*. Chroma, C*, was calculated from CIELAB color parameters as C* = $\sqrt{\left(a^{2}+b^{2}\right)}$, where a* represents the red–green axis and b* represents the yellow–blue axis. Each point represents one fruit color measurement. The half-violin plots show the distribution density of chroma values, and the box plots indicate the median and interquartile range. Whiskers represent the data range excluding extreme outliers. Different lowercase letters above the plots indicate significant differences among taxa according to one-way ANOVA followed by LSD post hoc multiple comparison test at *p* < 0.05. The overall ANOVA result was significant at *p* < 0.001. n = 90 fruits per taxon.

The chroma raincloud plot (Figure 5) visually reinforces the pronounced intertaxon gradient in color saturation. *I. rotunda* shows the highest C* values overall, whereas *I. latifolia* exhibits substantially lower C* values with a relatively tight distribution. *I. chinensis* and *I. cornuta* display compact violins with dense point clustering near their medians, indicating comparatively stable chroma within these taxa. By contrast, *I. rotunda* shows greater dispersion and a pronounced upper tail of high C* observations, suggesting higher within-taxon heterogeneity in saturation than the other taxa.

### 2.2. Electronic Nose Sensor Responses to Different Ilex Taxa

**Figure 6:**
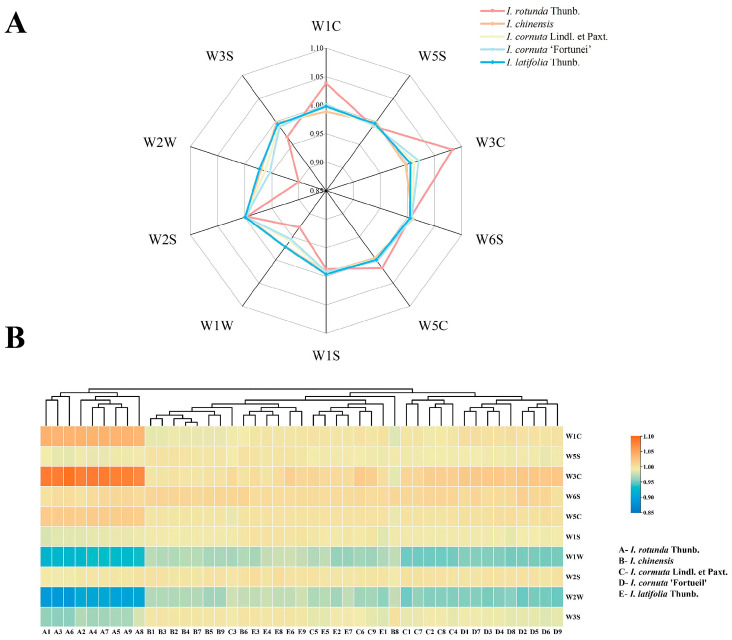
Electronic-nose response patterns of five *Ilex* taxa. (**A**) Radar plot showing the relative response intensities of the ten PEN3 electronic-nose sensors. Sensor codes are defined as follows: W1C, aromatic organic compounds; W5S, nitrogen oxides (broad-range sensitivity); W3C, ammonia and aromatic compounds; W6S, hydrogen; W5C, alkanes, aromatic compounds, and other non-polar organic compounds; W1S, methane and a broad range of organic compounds; W1W, sulfur-containing compounds and terpenes; W2S, alcohols and partially aromatic compounds; W2W, aromatic and sulfur-containing organic compounds; and W3S, methane/aliphatic compounds at relatively high concentrations. (**B**) Heatmap with hierarchical clustering based on the same sensor-response data. Taxon codes are as follows: A, *I. rotunda*; B, *I. chinensis*; C, *I. cornuta;* D, *I. cornuta* ‘Fortunei’; and E, *I. latifolia*. Numbers following each letter (for example, A1–A9) indicate biological replicates. Warmer colors indicate higher sensor responses, whereas cooler colors indicate lower sensor responses. Sensor descriptions follow the standard PEN3 sensor definitions.

To visualize intertaxon differences in volatile fingerprints at the sensor level, the steady-state responses of the 10 PEN3 MOS sensors were summarized in a radar plot (Figure 6A). The putative sensitivities of the sensors to different classes of odor-active volatiles are listed in Table 2. Among the examined taxa, *I. rotunda* exhibited the most distinctive sensor-response pattern. Specifically, *I. rotunda* showed pronounced responses for W3C and W1C, suggesting a greater relative contribution of aromatic and ammonia-related volatiles to its headspace profile. By contrast, W2W and W1W exhibited lower responses for *I. rotunda*, implying a lower relative contribution of these compound classes to its headspace profile.

In contrast, *I. chinensis*, *I. cornuta*, *I. cornuta* ‘Fortunei’, and *I. latifolia* showed relatively flat and largely overlapping profiles across most sensor axes. This similarity suggests broadly comparable volatile profiles among these taxa. Notably, the response curves of *I. cornuta* and its cultivar *I. cornuta* ‘Fortunei’ were nearly superimposable across all sensors, indicating limited divergence in their major volatile-response patterns. Taken together, these sensor-response fingerprints highlight *I. rotunda* as the most distinct taxon and provided the basis for subsequent chemometric discrimination.

To complement the radar plot, normalized multivariate sensor responses were visualized as a heatmap (Figure 6B), in which color intensity indicates relative response magnitude. In Figure 6B, columns represent samples and rows represent sensors; each cell shows the normalized response of a given sensor in a given sample. Warmer colors denote higher normalized responses, whereas cooler colors denote lower normalized responses. These results further identified *I. rotunda* as the most distinctive taxon in volatile-response space and showed that electronic-nose fingerprints can provide complementary descriptors for *Ilex* germplasm discrimination.

### 2.3. Principal Component Analysis of Electronic Nose Responses

**Figure 7:**
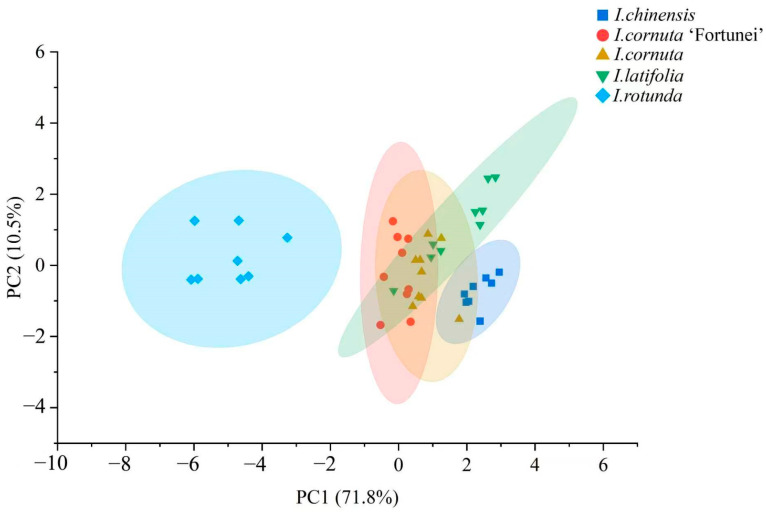
Principal component analysis of electronic-nose responses for discrimination of five *Ilex* taxa.

PCA was applied to the standardized electronic-nose sensor matrix to explore intertaxon variation in volatile fingerprints. The PCA score plot (Figure 7) revealed taxon-dependent clustering and separation patterns. *I. rotunda* was clearly separated from the other taxa, indicating pronounced differences in volatile-response fingerprints. Moreover, *I. rotunda* samples formed a tight cluster, indicating low within-taxon variation in volatile-response fingerprints. Although *I. chinensis* did not overlap with *I. rotunda*, its confidence ellipse partially overlapped with those of *I. cornuta* and *I. cornuta* ‘Fortunei’, indicating similar volatile-response fingerprints among these taxa. The confidence ellipses of *I. cornuta*, *I. cornuta* ‘Fortunei’, and *I. latifolia* showed substantial overlap, suggesting high similarity and limited discrimination by PCA alone. Within-group dispersion remained evident in the PCA space (Figure 7) [10]. These results indicate that PCA captured the major divergence represented by *I. rotunda*, whereas discrimination among the remaining taxa remained limited.

### 2.4. Linear Discriminant Analysis of Electronic Nose Responses

**Figure 8:**
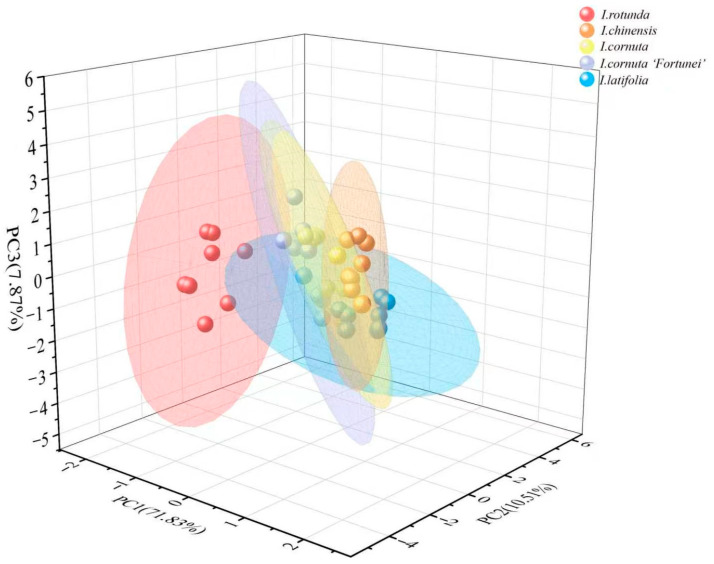
Linear discriminant analysis of electronic-nose responses for discrimination of five *Ilex* taxa.

LDA was applied to the electronic nose sensor response data from the five *Ilex* taxa. Figure 8 shows the LDA score plot based on electronic-nose volatile fingerprints for the five *Ilex* taxa. The first three linear discriminants (LD1, LD2, and LD3) accounted for approximately 100% of the discriminant variance (71.83%, 10.50%, and 17.87%, respectively), indicating that the three-dimensional space captured nearly all discriminatory information among taxa. Compared with PCA, LDA further improved separation between *I. rotunda* and the remaining taxa. In addition, *I. rotunda* samples formed a tight cluster within the confidence ellipse, indicating high within-taxon consistency in volatile fingerprints. These results show that supervised analysis of electronic-nose fingerprints enhanced the discrimination of closely related *Ilex* taxa.

### 2.5. OPLS-DA Model for Targeted Discrimination of Ilex rotunda

**Figure 9:**
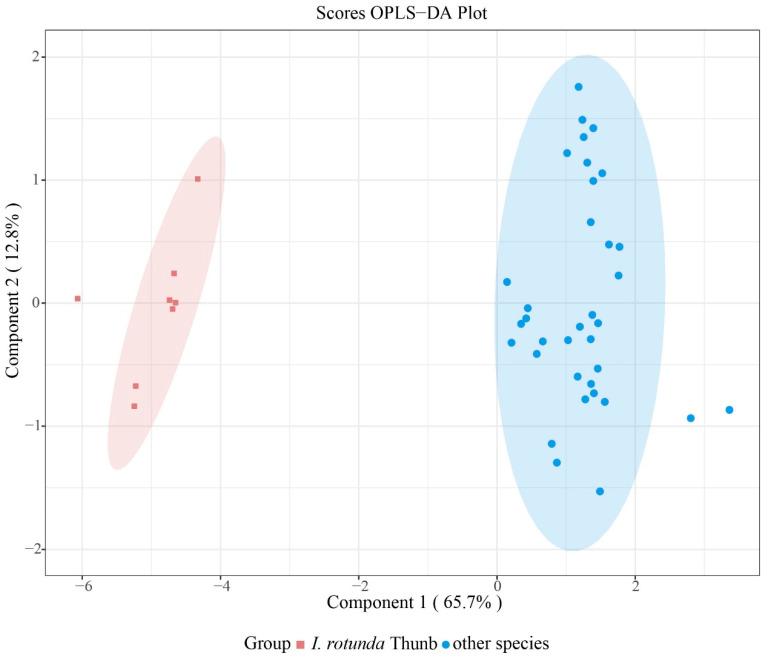
OPLS-DA score plot for discrimination of *Ilex* rotunda from the other four *Ilex* taxa based on electronic nose responses. Samples formed two distinct clusters primarily along the predictive component p1 (R^2^X = 0.657), whereas the orthogonal component o1 accounted for unrelated variation (R^2^X = 0.128). The separation indicates stable multivariate differences in electronic-nose sensor fingerprints between *I. rotunda* and the non-target taxa.

To develop a targeted identification model for *Ilex rotunda*, OPLS-DA was applied to the preprocessed electronic-nose response matrix using a binary class design (*I. rotunda* vs. non-target taxa). The OPLS-DA score plot showed clear separation between *I. rotunda* and the remaining four taxa, primarily along the predictive component p1 (Figure 9). The *I. rotunda* samples formed a compact cluster, indicating relatively low within-class variation in volatile fingerprints. By contrast, the non-target group showed greater dispersion, which was expected because it comprised four different taxa. Nevertheless, the two classes remained clearly separated, demonstrating that *I. rotunda* possessed a distinctive multivariate sensor-response pattern.

**Figure 10:**
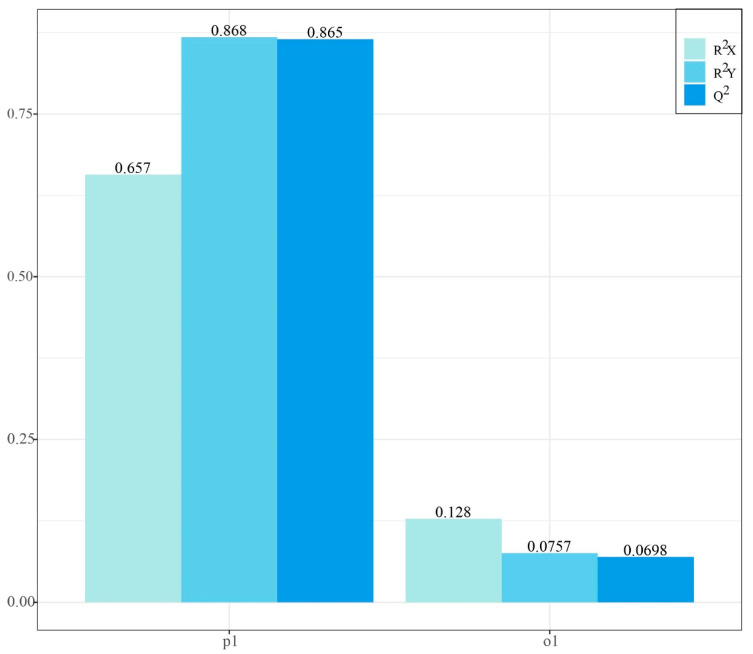
Cross-validation summary plot of the OPLS-DA model for discrimination of *Ilex rotunda* from the non-target taxa. The model comprised one predictive component (p1) and one orthogonal component (o1). For p1, R^2^X = 0.657, R^2^Y = 0.868, and Q^2^ = 0.865; for o1, R^2^X = 0.128, R^2^Y = 0.0757, and Q^2^ = 0.0698. These results indicate strong explained variance and high cross-validated predictive performance.

The cross-validation summary further supported the discriminatory performance of the model (Figure 10). The model comprised one predictive component (p1) and one orthogonal component (o1). The predictive component explained a substantial proportion of both X and Y variation (R^2^X = 0.657, R^2^Y = 0.868, Q^2^ = 0.865), whereas the orthogonal component accounted for a smaller proportion of structured variation unrelated to class separation (R^2^X = 0.128, R^2^Y = 0.0757, Q^2^ = 0.0698). These results indicate that class discrimination was driven mainly by the predictive component and that the model showed strong cross-validated predictive ability.

The OPLS-DA model included one predictive component (p1) and one orthogonal component (o1). Model performance was assessed using R^2^X, R^2^Y, and cross-validated predictive ability (Q^2^). Model robustness and potential overfitting were further assessed by permutation testing (n = 200). Q^2^ was obtained by cross-validation as defined below [11]:

$$R^{2}X=1-\frac{\mathrm{SS}\left(E\right)}{\mathrm{SS}\left(X_{\mathrm{cnee}}\right)},R^{2}Y=1-\frac{\mathrm{SS}\left(F\right)}{\mathrm{SS}\left(Y_{\mathrm{cnee}}\right)}$$

$$Q^{2}=1-\frac{\mathrm{SS}\left(F_{C}\right)}{\mathrm{SS}\left(Y_{\mathrm{cnee}}\right)}$$

**Figure 11:**
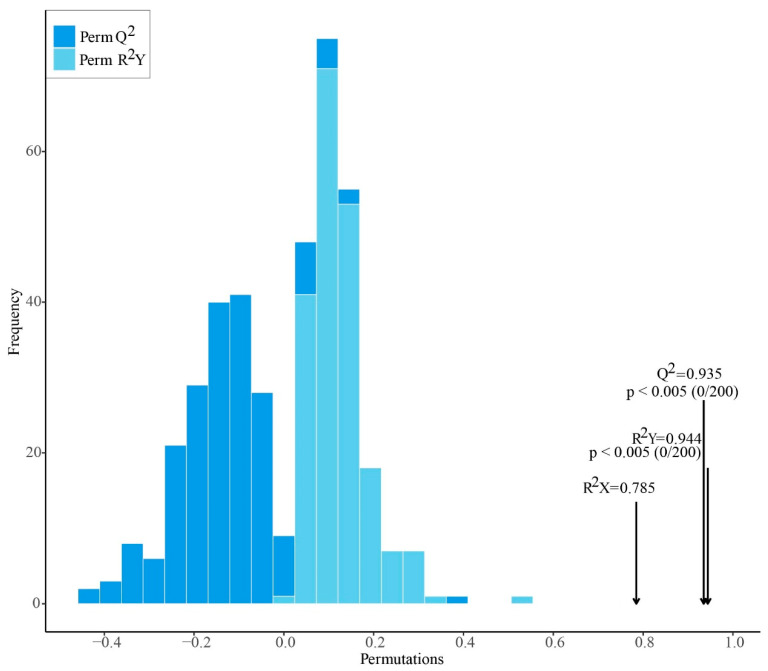
Distribution of permuted R^2^Y and Q^2^ values from 200 permutation tests of the OPLS-DA model for discrimination of *Ilex rotunda* from the other *Ilex* taxa. Histograms show the frequency distributions of the permuted models, and arrows indicate the original model values (R^2^X = 0.785, R^2^Y = 0.944, and Q^2^ = 0.935). The original model outperformed all permuted models (*p* < 0.005), supporting model robustness.

Model robustness was further evaluated by permutation testing. The distribution plot of 200 permuted models showed that the original model values for R^2^Y and Q^2^ were clearly separated from the distributions of the permuted models (Figure 11). The original model (R^2^Y = 0.944, Q^2^ = 0.935) outperformed all permuted models (*p* < 0.005), indicating that the observed class separation was unlikely to have arisen by chance. In addition, the original model showed a relatively high R^2^X value (0.785), consistent with good overall explanatory ability.

**Figure 12:**
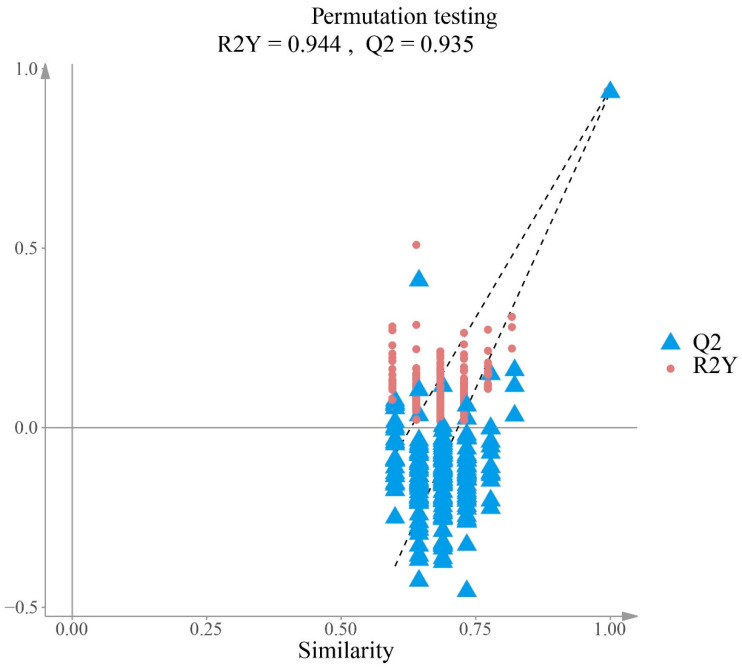
Permutation test plot of the OPLS-DA model for discrimination of *Ilex* rotunda from the other *Ilex* taxa (n = 200). Blue triangles and red circles represent Q^2^ and R^2^Y values, respectively, for permuted models at different levels of class-label similarity. Dashed regression lines summarize the trends of Q^2^ and R^2^Y across permutations. The original model (R^2^Y = 0.944, Q^2^ = 0.935) was clearly separated from the permuted models, indicating good predictive performance.

The permutation trend plot provided additional support for model validity (Figure 12). Across decreasing levels of class-label similarity, the permuted models consistently exhibited markedly lower R^2^Y and Q^2^ values than the original model. In particular, many permuted Q^2^ values were below zero, indicating poor predictive performance under random class assignment. Together with the clear separation of the original model from the permuted models, these results suggest that the OPLS-DA classification was robust and showed a low risk of overfitting.

VIP was used to evaluate the contribution of each sensor to class discrimination in the OPLS-DA model. Variables with VIP > 1 were considered major contributors. VIP values were calculated as follows [12]:

$${\mathrm{VIP}}_{j}=\sqrt{\frac{p}{h}\sum_{f=1}^{h}\left(w_{jf}^{2}\cdot \mathrm{SS}\left(T_{f}\right)\right)/\sum_{f=1}^{h}\mathrm{SS}\left(T_{f}\right)}$$

**Figure 13:**
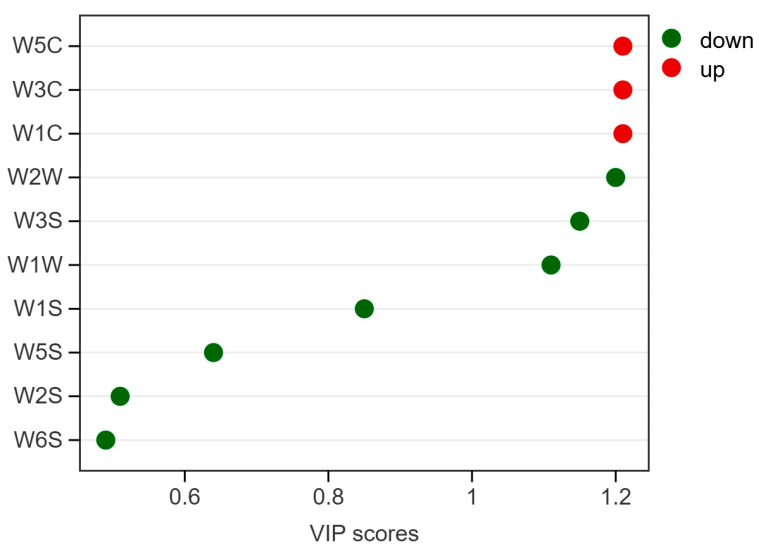
Variable importance in projection (VIP) plot of the OPLS-DA model for discrimination of *Ilex rotunda*. Variables are ranked according to their VIP scores. Sensors with VIP > 1 (e.g., W5C, W3C, W1C, W2W, W3S, and W1W) were identified as major contributors to class discrimination and may serve as candidate marker features for targeted identification.

To identify the variables contributing most strongly to class discrimination, variable importance in projection (VIP) scores were examined (Figure 13). Six sensors showed VIP values greater than 1 and were therefore considered the major contributors to discrimination: W5C, W3C, W1C, W2W, W3S, and W1W. The remaining sensors (W1S, W5S, W2S, and W6S) showed lower contributions. These findings indicate that discrimination of *I. rotunda* depended on a multivariate sensor signature rather than on any single sensor response. The high-VIP sensors may therefore serve as candidate marker features for targeted identification in future studies.

Overall, the OPLS-DA results demonstrate that *I. rotunda* can be reliably distinguished from the other four *Ilex* taxa based on electronic-nose responses. The combination of clear class separation, strong cross-validated predictive performance, favorable permutation test results, and informative VIP profiles supports the use of this approach as a rapid and non-destructive tool for targeted germplasm identification.

**Figure 14:**
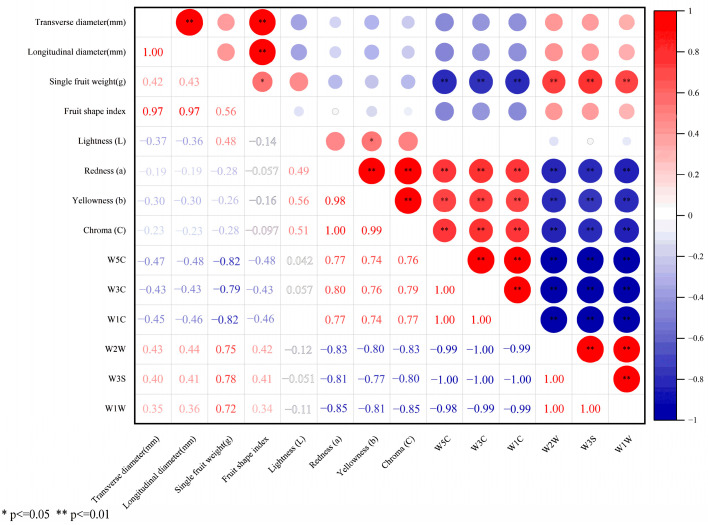
Pearson correlation matrix between fruit morphometric/colorimetric traits and key electronic-nose sensors (VIP > 1, identified by OPLS-DA) in five *Ilex* taxa. The lower triangle shows exact correlation coefficients; the upper triangle visualizes correlations via circle plots, with color and size representing correlation direction and strength, respectively. Significance levels: * *p* ≤ 0.05; ** *p* ≤ 0.01.

To further clarify the biological relevance of the key discriminatory sensors (VIP > 1) identified by the OPLS-DA model, we performed Pearson correlation analysis between their response values and fruit phenotypic traits (Figure 14). The results showed that the sensors most responsible for the discrimination of *I. rotunda* were closely associated mainly with peel color traits, and to a lesser extent with single-fruit weight. Specifically, W5C, W3C, and W1C, which contributed positively to the separation of *I. rotunda*, were significantly and positively correlated with fruit redness (a*), yellowness (b*), and chroma (C*) (r = 0.74–0.80, *p* < 0.01). In contrast, W2W, W3S, and W1W, which contributed negatively to *I. rotunda* discrimination, were significantly and negatively correlated with these color variables (r = −0.80 to −0.85, *p* < 0.01). This correlation pattern was consistent with the phenotypic profile of *I. rotunda*, which exhibited the highest redness and chroma values among the five taxa, thereby supporting the reliability of the electronic-nose-based discrimination model. By contrast, most fruit size- and shape-related traits showed weak or non-significant correlations with sensor responses, indicating that the electronic nose primarily captures variation associated with color-related volatile differences rather than gross fruit morphology. These results help define the practical scope of the method, suggesting that it is more suitable for distinguishing taxa with divergent volatile and peel-color characteristics than for evaluating fruit morphological traits alone.

## 3. Discussion

The present results show that fruit morphometric and colorimetric traits provide useful information for distinguishing the five examined *Ilex* taxa, while E-nose profiling adds complementary discriminatory power beyond visible fruit phenotype alone [13]. This supports the view that integrated fruit-based characterization can improve the identification of closely related *Ilex* materials and provide a practical supplementary tool for germplasm evaluation [14].

Among the measured traits, fruit size and fresh weight were especially informative for *I. cornuta*, which produced the largest and heaviest fruits. These features are relevant not only for preliminary recognition but also for ornamental evaluation, as larger fruits generally contribute more strongly to visual display. In contrast, *I. chinensis* had the highest fruit shape index (FSI), corresponding to the most elongated fruits; this visually intuitive trait may help separate it from taxa with nearly round fruits [15]. Color traits were particularly informative for *I. rotunda*, which showed the highest redness (a*) and chroma (C*) values, indicating the brightest and most saturated red peel [16]. Such parameters are especially meaningful in *Ilex*, whose ornamental value is closely associated with winter fruit display. *I. latifolia*, by contrast, was characterized by a combination of relatively low fruit weight, low FSI, and low L*, a*, b*, and C* values, indicating comparatively small, nearly round fruits with darker and less saturated coloration. These findings suggest that visible fruit traits have practical value for preliminary discrimination and utilization-oriented evaluation, although the similarity between *I. cornuta* and *I. cornuta* ‘Fortunei’ also indicates that external phenotype alone may be insufficient when closely related materials overlap in phenotypic range [17].

E-nose profiling helped address this limitation by providing an additional layer of volatile-response information [18]. Previous applications of E-nose technology in *Ilex* have focused mainly on tea-quality evaluation and postharvest studies, whereas its taxonomic use has remained limited [19]. In this study, chemometric analysis of E-nose responses improved overall separation among taxa, supporting the advantage of combining morphology, color, and odor-response data rather than relying on any single category of traits. The value of E-nose profiling here is primarily methodological: it strengthens accession verification when visible traits are insufficient. Compared with GC-MS, E-nose analysis is rapid, non-destructive, and relatively low-cost, making it suitable for preliminary screening or high-throughput germplasm comparison [6]. At the same time, the results should be interpreted as sensor-response fingerprints rather than direct chemical identifications, and fruit maturity should be carefully standardized because volatile release may vary with developmental stage.

Several limitations should be noted. Only five representative taxa from eastern China were included, so the broader applicability of the framework across the genus remains to be tested. In addition, because the present approach is mainly fruit-based, it does not fully resolve year-round identification in this evergreen genus. In practice, *Ilex* taxa are often encountered outside the fruiting season, when identification relies largely on leaf traits; however, leaf shape and size may vary among taxa and overlap among closely related species or cultivated forms. Thus, fruit traits should be regarded as informative supplementary descriptors rather than a complete solution for evergreen germplasm identification, and future studies should integrate fruit- and leaf-based characters, including leaf volatile signals, while further standardizing sampling stage [20]. Moreover, the present study focused on phenotypic discrimination rather than on the anatomical, biochemical, or molecular basis of trait variation. Future work should therefore expand taxon sampling and combine E-nose profiling with GC-MS, metabolomics, histology, and, where appropriate, transcriptomic analyses.

Overall, the five taxa differed significantly in fruit morphometric, colorimetric, and odor-response traits, and the integrated dataset provided stronger discrimination than visible traits alone. Fruit morphology, colorimetry, and volatile-response profiling therefore provide a practical supplementary framework for *Ilex* germplasm characterization, documentation, and utilization-oriented evaluation.

## 4. Materials and Methods

### 4.1. Plant Materials, Study Site, and Instruments

#### 4.1.1. Plant Materials and Study Site

Fruits of five *Ilex* taxa (*I. rotunda*, *I. chinensis*, *I. cornuta*, *I. cornuta* ‘Fortunei’, and *I. latifolia*) were collected from the campus of Nanjing Forestry University (Xuanwu District, Nanjing, Jiangsu Province, China; 32.08° N, 118.82° E). All sampled mother plants have been archived with unique, traceable germplasm accession numbers in the nursery: *I. rotunda* (NFU-IL-2008-007), *I. chinensis* (NFU-IL-2008-012), *I. cornuta* (NFU-IL-2007-023), *I. cornuta* ‘Fortunei’ (NFU-IL-2007-028), and *I. latifolia* (NFU-IL-2008-015).The study site is located in a subtropical monsoon climate zone with four distinct seasons, a mean annual temperature of 15.4 °C, annual precipitation of 1000–1100 mm, and an annual frost-free period of approximately 237 days. The five taxa were selected as representative *Ilex* germplasm resources from eastern China and were maintained under uniform site conditions and consistent cultivation management to minimize environmental heterogeneity. Fruits of each taxon were collected from three healthy, adult individual plants with the above fixed germplasm accession numbers, with fruits sampled randomly at the fully mature stage from accessible fruiting branches. Formal voucher specimens for all five studied taxa were collected synchronously during sampling, taxonomically authenticated by the corresponding author Dr. Ye Peng, and deposited in the Herbarium of College of Life Sciences, Nanjing Forestry University (NFU, Nanjing, China). The voucher numbers for each taxon are as follows: *I. rotunda* (NFU202601001), *I. chinensis* (NFU202601002), *I. cornuta* (NFU202601003), *I. cornuta* ‘Fortunei’ (NFU202601004), and *I. latifolia* (NFU202601005).

#### 4.1.2. Sampling Procedure

Fruit samples were collected at the fully mature stage in January 2026. For each taxon, fruits were harvested from three healthy adult plants. For morphological and colorimetric analyses, more than 200 fruits per taxon were initially collected. After screening for uniform maturity and the absence of visible disease symptoms, pest damage, and mechanical injury, 90 fruits per taxon were retained for subsequent analysis. The selected fruits were then divided into three biological replicates, with 30 fruits per replicate.

For electronic nose analysis, nine biological replicates were prepared for each taxon, and each replicate consisted of 2.0 g of fruit material pooled from the three sampled plants. Only healthy fruits free from visible defects were included in all analyses.

#### 4.1.3. Instruments

A stereomicroscope (SMZ1270, Nikon, Tokyo, Japan) was used to acquire representative images of mature fruits for visual documentation of fruit size, shape, and peel coloration. Volatile fingerprints were acquired using a PEN3 portable electronic nose equipped with 10 metal oxide semiconductor (MOS) sensors (AIRSENSE Analytics GmbH, Schwerin, Germany). Fruit color was measured using a TS7010 colorimeter (Shenzhen Threenh Technology Co., Ltd., Shenzhen, China). Single-fruit fresh weight was measured using an electronic balance (JA5003; readability: 0.001 g; Shanghai Liangping Instruments Co., Ltd., Shanghai, China). Fruit longitudinal and transverse diameters were measured using digital calipers.

### 4.2. Experimental Procedures

#### 4.2.1. Electronic Nose Measurement

To characterize volatile-response patterns among the five *Ilex* taxa, mature fruits collected from the three sampled plants of each taxon were pooled to obtain composite samples, following previous studies [21]. For each replicate, a 2.0 g aliquot of fruit material was placed in a 50 mL headspace vial, sealed immediately [22], and incubated for 30 min to allow headspace equilibration [23]. Headspace volatiles were then analyzed using the PEN3 electronic nose. Only mature fruits were included in the present study. Unripe and overripe fruits were not sampled because the aim was to evaluate taxon discrimination using mature fruits relevant to routine germplasm characterization. Ripeness was standardized operationally at the mature stage rather than quantified using physiological indices.

Preliminary tests indicated that the sensor signals stabilized after approximately 70 s and reached a stable plateau at 78 s. Therefore, the response values recorded at 78 s were used as the characteristic variables for subsequent analyses [17]. The instrument was operated under the following conditions: flushing time, 80 s; zero-adjustment time, 5 s; sample preparation time, 5 s; injection mode, headspace sampling; injection time, 80 s; and carrier gas, dry air at a flow rate of 300 mL min^−1^ [18]. Each taxon was analyzed using nine biological replicates to ensure robust characterization of the volatile-response profiles.

#### 4.2.2. Determination of Fruit Traits

Healthy mature fruits selected as described in Section 2.1.2 were used for trait measurements.

Single-fruit fresh weight (FW, g) was measured using a JA5003 electronic balance. Fruit longitudinal diameter (LD, mm) and transverse diameter (TD, mm) were measured using digital calipers. The fruit shape index (FSI) was calculated as follows [24]:

$$\mathrm{FSI}=\frac{\mathrm{LD}}{\mathrm{TD}}$$

Fruit peel color was measured using a TS7010 colorimeter on smooth and intact areas free from blemishes and wrinkles, with the measuring aperture held flush against the fruit surface [25]. The instrument was calibrated against black and white reference standards before measurement. Color was recorded in the CIELAB system as L* (0 = black, 100 = white), a* (negative values indicate green and positive values indicate red), and b* (negative values indicate blue and positive values indicate yellow). For each biological replicate, the mean L*, a*, and b* values were calculated from 30 fruits, resulting in three replicate means for each taxon. Chroma (C*) was calculated according to McGuire et al. [19]:

$$C^{*}=\sqrt{{a^{*}}^{2}+{b^{*}}^{2}}$$

For visualization of fruit color differences among taxa, the mean L*, a*, and b* values were converted into RGB values using Python 3.9 [20]. Representative mature fruits of each taxon were also photographed under a stereomicroscope in lateral and apical views against a uniform background with a scale bar for visual comparison of fruit size, shape, and peel coloration.

### 4.3. Data Processing and Statistical Analysis

Raw data were organized in Excel 2019 prior to statistical analysis. To improve comparability among sensors and eliminate scale effects in the electronic nose dataset, the sensor response matrix was standardized using Z-score normalization (autoscaling) prior to multivariate analysis [26].

In the equation below, x_ij_ represents the original response of sensor j in sample i and μ_j_ and σ_j_ represent the mean and standard deviation of sensor j across all samples, respectively:

$$z_{ij}=\frac{x_{ij}-\mu_{j}}{\sigma_{j}}$$

The normalized electronic nose data were then subjected to principal component analysis (PCA) and linear discriminant analysis (LDA) using WinMuster (V.1.6.2) software (AIRSENSE Analytics GmbH, Germany) [27].

For fruit phenotypic traits, intertaxon differences were evaluated using one-way analysis of variance (ANOVA), followed by the least significant difference (LSD) test for pairwise comparisons at *p* < 0.05 [28]. All ANOVA and LSD analyses were performed in R version 4.2.1 (R Foundation for Statistical Computing, Vienna, Austria) [29]. One-way ANOVA was fitted with the aov() function from the base R stats package, and LSD post hoc tests were conducted using the LSD.test() function from the agricolae package [30], which automatically generates compact letter displays to identify statistically homogeneous groups. Descriptive statistics and pre-analysis data processing were also completed in the R environment using base R functions.

## 5. Conclusions

This study demonstrates that an integrated, non-destructive framework combining fruit morphometric traits, CIELAB color parameters, and PEN3 electronic-nose profiling effectively discriminates five representative *Ilex* taxa from eastern China. The findings reject the null hypotheses and support the hypothesis that fruit volatile profiles, as captured by E-nose, can provide informative markers for distinguishing closely related *Ilex* taxa. Fruit morphology and color captured substantial intertaxon variation in visible phenotype, while E-nose profiling contributed complementary odor-response information, confirming the value of this integrated approach for germplasm characterization and utilization-oriented evaluation.

Among the examined taxa, *I. cornuta* was characterized by the largest and heaviest fruits, *I. chinensis* by the highest fruit shape index and the most elongated fruits, and *I. rotunda* by the highest redness and chroma values, corresponding to the brightest and most saturated red peel. *I. latifolia* was distinguished by a characteristic combination of low single-fruit weight, relatively low fruit shape index, and comparatively low L*, a*, b*, and C* values, indicating relatively small, nearly round fruits with darker and less saturated coloration. Electronic-nose profiling further strengthened taxon discrimination, identifying *I. rotunda* as the most distinct taxon in volatile-response space, whereas *I. cornuta* and *I. cornuta* ‘Fortunei’ remained the most similar pair.

Beyond taxon discrimination, this study also indicates that several fruit traits can serve as practically informative supplementary descriptors for germplasm documentation and utilization. Fruit size and weight were especially relevant to ornamental prominence, fruit shape index to the evaluation of distinctive fruit form, and redness and chroma to the objective assessment of fruit display quality. Taken together, these findings suggest that the combined use of morphology, colorimetry, and volatile-response profiling can complement traditional classification, support accession verification and germplasm documentation, and provide a practical basis for utilization-oriented evaluation and parent selection in future *Ilex* breeding and horticultural improvement.

## Figures and Tables

**Table 1:** Fruit morphometric and colorimetric descriptors of five *Ilex* taxa used for germplasm characterization.

	*I. rotunda*	*I. chinensis*	*I. cornuta*	*I. cornuta* ‘Fortunei’	*I*. *latifolia*	*p*-Value
Transverse diameter (TD) (mm)	5.64 ± 0.39 ^d^	7.09 ± 0.48 ^b^	7.62 ± 0.51 ^a^	6.79 ± 0.43 ^c^	6.84 ± 0.45 ^c^	<0.001
Longitudinal diameter (LD) (mm)	5.86 ± 0.41 ^e^	8.50 ± 0.57 ^b^	8.83 ± 0.62 ^a^	7.91 ± 0.53 ^c^	7.08 ± 0.49 ^d^	<0.001
Single fruit weight (FW) (g)	0.203 ± 0.022 ^c^	0.231 ± 0.028 ^b^	0.313 ± 0.035 ^a^	0.196 ± 0.021 ^c^	0.183 ± 0.019 ^d^	<0.001
Fruit shape index (FSI)	1.038 ± 0.049 ^c^	1.200 ± 0.062 ^a^	1.161 ± 0.055 ^b^	1.169 ± 0.058 ^b^	1.036 ± 0.047 ^c^	<0.001
Lightness (L)	41.82 ± 2.21 ^c^	45.26 ± 2.31 ^a^	42.58 ± 2.25 ^b^	43.26 ± 2.19 ^b^	40.55 ± 2.18 ^d^	<0.001
Redness (a)	52.90 ± 3.25 ^a^	46.89 ± 2.41 ^c^	48.23 ± 2.36 ^b^	45.32 ± 2.38 ^d^	35.92 ± 2.87 ^e^	<0.001
Yellowness (b)	33.24 ± 2.98 ^a^	29.35 ± 2.23 ^b^	28.86 ± 2.15 ^b^	27.68 ± 2.19 ^c^	22.16 ± 2.53 ^d^	<0.001
Chroma (C)	62.70 ± 3.52 ^a^	55.83 ± 3.09 ^b^	56.28 ± 3.12 ^b^	52.75 ± 3.15 ^c^	42.21 ± 3.15 ^d^	<0.001

Values are presented as mean ± SD. Different lowercase letters within a row indicate significant differences among taxa (LSD post hoc test, *p* < 0.05). All traits showed significant intertaxon differences (one-way ANOVA, *p* < 0.001).

**Table 2:** Types and performance of electronic nose sensors.

Number	Sensor	Response-Sensitive Flavor Compound Categories
1	W1C	Aromatic compounds (benzenes)
2	W5S	Nitrogen oxides
3	W3C	Aromatic compounds; ammonia-related compounds
4	W6S	Hydrogen-containing compound
5	W5C	Short-chain alkanes; aromatic compounds
6	W1S	Alkanes
7	W1W	Sulfur-containing organic compounds
8	W2S	Alcohols, aldehydes and ketones
9	W2W	Sulfur-containing, chlorine-containing organic compounds
10	W3S	Aliphatic alkane compounds

## Data Availability

The data presented in this study are available from the corresponding author upon reasonable request.
